# Missense and Nonsense Mutations of P53 Gene in Patients with Colorectal Adenocarcinoma in Isfahan, Central Iran

**Published:** 2011-03-01

**Authors:** R Golmohammadi, M J Namazi, M Nikbakht, M Salehi

**Affiliations:** 1 Faculty of Medicine, Sabzevar University of Medical Sciences, Sabzevar, Iran; 2 Faculty of Medicine, Isfahan University of Medical Sciences, Isfahan, Iran

**Keywords:** Colorectal cancer, Missense, Nonsense, Mutations, P53 gene

Dear Editor,

The colorectal cancer is the forth prevalent cancer worldwide. The incidence and mortality of colorectal cancer (CRC) in men and women has been estimated more than 1.233 million cases and nearly 610000 deaths annually respectively.[1] It is also the forth prevalent cancer in Iran.[2] The CRC occurrence is correlated with genetic and non-genetic factors such as missense and non-missense mutations in a tumor suppressor P53 gene.[3] It is known that most of point mutations happen in exons 5 to 8 of the P53 gene with different prevalence in different regions.[4] Many studies showed that the frequency of P53 mutation is between 43% and 50% in CRC patients.[5] Therefore, detection of such mutations would be very important for any successful chemo-radiotherapy measure. The present study aimed to detect and characterize different mutations in exons 5 and 6 of the P53 gene in patients with CRC in Isfahan, central Iran.

Sixty one 62.4±9 years patients who were admitted in some hospitals in Isfahan mainly Al-Z hra Hospital were enrolled. An ethical written agreement was provided and their samples were blindly used in the examinations. Both healthy, as control, and tumoral tissues, as samples, were taken and their DNA samples were obtained using phenol chloroform. The exons 5 and 6 of the P53 gene were amplified by polymerase chain reaction (PCR) using following designed primers: (5´ TGTTCACTTGTGCCCTGACT 3´, 5´ GGAGGGCCACTGACAACCA3´).

The length of exons 5 and 6 were designed to have 489bp. [6] Single stranded conformation polymorphism (SSCP) analysis and subsequently sequencing was performed for each PCR and electrophoresis product. To determine any significant difference, data were analyzed using Fisher Exact test.

Sixty one samples were used of which 15 (25%) were females and 46 (75%) were males. Fourteen samples were positive (12 males and 2 females) among them, 3 cases had more than one mutation resulting in 19 point mutations of which 17 were missense and 2 were nonsense. There was a significant (p=0.032) diference between the stage of tumor and the presence of the mutation. However, no significant difference was observed between mutations and their locations in distal or proximal colon. There was a consistency between the results of sequencing and PCR-SSCP examination. Yamashita et al. found similar results in 6 patients with CRC out of 20 subjects in Sindaie, Japan. [7] However, Pan et al. reported only one mutation out of 97 cases with rectal carcinoma. 6 Similar to our results, most studies showed more frequent mutations in proximal colon compared with distal colon.[8] This may be due to presence of more concentrated toxic substances in the distal region based on dietary habits having more fast foods.[9]

To the best of our knowledge, our findings are the first report for the presence of point mutation in exons 5 and 6 in CRC patients in Isfahan, central Iran. We also observed higher rate of mutations in males compared to females. As some other researchers have also suggested, we can assume that males are more exposed to the risk factors of CRC.[10] Further studies are required with a larger sample size to determine the different risk factors, possible interactions between suppressor genes and their related markers in CRC patients to help to find more efficient preventive and therapeutic strategies.
